# Determinants of adherence to clinic appointments among tuberculosis and HIV co-infected individuals attending care at Helen Joseph Hospital, Johannesburg, South Africa

**DOI:** 10.11604/pamj.2020.37.118.23523

**Published:** 2020-10-05

**Authors:** Ruvimbo Barbara Claire Nhandara, Birhanu Teshome Ayele, Lovemore Nyasha Sigwadhi, Lovelyn Uzoma Ozougwu, Peter Suwirakwenda Nyasulu

**Affiliations:** 1 Division of Epidemiology and Biostatistics, Faculty of Medicine and Health Sciences, Stellenbosch University, Cape Town, South Africa,; 2 Division of Epidemiology and Biostatistics, Faculty of Health Sciences, School of Public Health, University of the Witwatersrand, Johannesburg, South Africa

**Keywords:** Adherence, tuberculosis, HIV/AIDS, co-infection, Johannesburg, South Africa, treatment duration, Helen Joseph Hospital, clinic appointments

## Abstract

**Introduction:**

tuberculosis (TB) is one of the leading causes of morbidity and mortality among people living with HIV/AIDS. The growing burden of TB/HIV co-infection continues to strain the healthcare system due to association with long duration of treatment. This is a catalyst for poor adherence to clinic appointments, which results in poor treatment adherence and patient outcome. This study evaluated the factors associated with adherence to clinic appointments among TB/HIV co-infected patients in Johannesburg, South Africa.

**Methods:**

this was a cross-sectional study that involved 10427 patients ≥18 years of age with HIV infection and co-infected with TB. We used a proxy measure “md clinic appointments” to assess adherence, then multivariable logistic regression to evaluate factors associated with adherence.

**Results:**

one thousand, five hundred and twenty-eight patients were co-infected with TB, of these, 17.4% attained good adherence. Patients with TB/HIV co-infection who were on treatment for a longer period were less likely to adhere to clinic appointments (AOR: 0.98 95% CI: 0.97, 0.99).

**Conclusion:**

duration on treatment among TB/HIV co-infected patients is associated with adherence to clinic appointments. It is therefore vital to reinforce public health interventions that would enhance sustained adherence to clinic appointments and mitigate its impact on treatment adherence and patient outcome.

## Introduction

Tuberculosis (TB) remains a major public health challenge, which has been compounded over the past four decades by the Human Immunodeficiency Virus (HIV) epidemic [1]. As the prevalence of HIV continues to increase, the proportion of individuals in the population with compromised immune system also increased leading to an increase in TB cases [1]. In 2014, TB was the leading infectious cause of death [2] and the most common opportunistic infection among people living with HIV infection (PLWH) [3-5]. Notwithstanding, TB has been over-diagnosed in PLWH due to its close similarity in clinical presentation with other pathogens such as *Pneumocystis jirovecii* and Norcadia [6]. In the presence of a weakened immune system, *Mycobacterium tuberculosis* progressively multiplies and causes TB. As such, an individual with TB/HIV co-infection has a higher likelihood of developing active TB [7,8]. The TB/HIV co-infection has significantly increased the burden of infectious diseases worldwide [3].

In 2017, an estimated 10 million people developed active TB of whom 9% were PLWH. The World Health Organization estimates that one third of the estimated 36.9 million PLWH were co-infected with TB. An estimated 1.3 million people died of TB with approximately 300,000 TB related deaths occurring among PLWH [9,10]. This has overstretched the healthcare system due to association with lengthy treatment regimen that prolongs treatment duration. Such a scenario is a precipitating factor for poor treatment adherence, which is a major public health challenge due to its propensity to drive anti-tuberculous drug resistance. This is so because missing scheduled clinic appointments among patients with dual infections is a catalyst for poor treatment adherence and patient outcomes [4,11-13]. This therapeutic challenge has often led to treatment interruption for both HIV/AIDS and TB medication leading to prolonged duration on treatment and occurrences of drug resistance [6,14].

South Africa is among the world´s most affected with TB/HIV co-infection contributing to an estimated 20% of the global burden of TB/HIV co-infections [15]. This has created a scenario where a large proportion of TB/HIV co-infected individuals in South Africa require concurrent treatment for TB and HIV/AIDS [16]. The dual infection has had a compounding effect on sustaining treatment adherence among affected patients. It is common knowledge that where TB treatment adherence is maximally achieved, TB is usually completely cured [17]. The same applies to HIV infection where viral suppression is achieved and sustained with acceptable level of adherence to Antiretroviral Therapy (ART). This fundamental paradigm shift in medication adherence would have a resultant effect in reduction of HIV transmission and would prolong and improve the quality of life of those infected [18,19]. Despite the advances in treatment availability and accessibility, TB/HIV co-infection has remained a compounded public health challenge [20] requiring innovative approach specifically in South Africa, a high burden country. Therefore, assessing the determinants of adherence to clinic appointments among TB/HIV infected patients is critical to help identify effective health system interventions that could be used to support individuals taking both TB medication and ART for improved treatment outcomes.

## Methods

**Study design:** the study used a retrospective record review of patients with TB/HIV co-infection to access adherence to ART/TB clinic appointments.

**Study site:** this study was conducted at Themba Lethu Clinic in October 2016. The clinic is located at Helen Joseph Hospital, Westdene, in Johannesburg metropolitan area. It provides HIV/AIDS and TB related treatment, care and support services.

**Figure 1:**
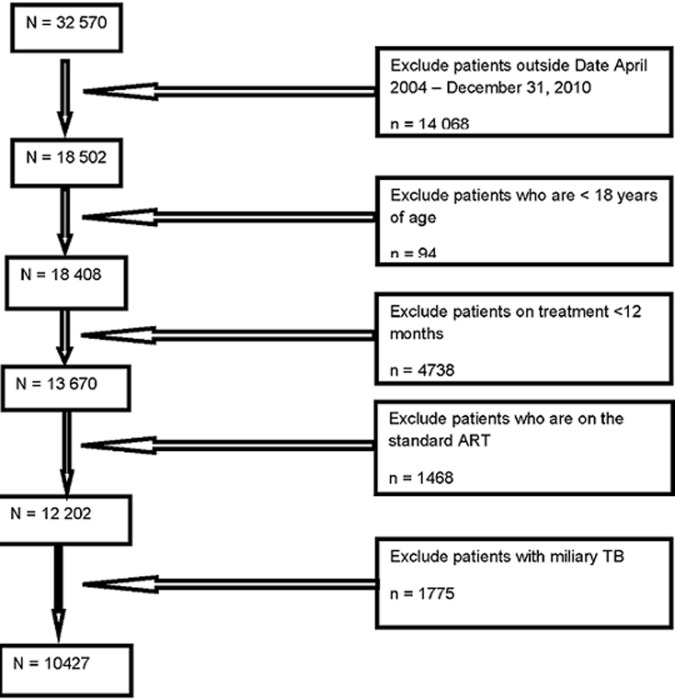
flow diagram showing number included in the final analysis

**Sampling and sample size:** Themba Lethu Clinic had a total population of 32,570 registered patients that were followed up from April 2004 to April 2010. Patients aged ≥18 years, HIV positive, with a TB diagnosis, ART naïve at the time of registration, on standard first line ART, who were within a minimum of 12 months´ follow-up and had initiated treatment between April 2004 and April 2010, were included. Patients with extra-pulmonary TB were excluded from the study. A total of 10,427 individuals met the inclusion criteria, of which 6,781 (65%) were females and 3,646 (35%) were males (Figure 1).

**Data collection:** data were extracted from ´therapy edge´ database that houses HIV patients´ treatment records at Themba Lethu Clinic. The following variables were extracted: age, sex, education, employment status, alcohol consumption, smoking status, TB co-infection at baseline, baseline CD4 count, haemoglobin level, WHO baseline, body mass index (BMI), months on individual treatment and months on treatment by co-infection among patients with TB/HIV co-infection.

**Variable measurements:** adherence was measured by assessing the number of missed clinic appointments. It was categorised into two groups “good adherence”, those who attained greater than 90% of appointment visits and “poor adherence”, those who achieved less than 90% of scheduled clinic appointments [19,20]. The exposure variables were: demographic (sex, age, marital status); socio-economic (source of income, educational level); laboratory (AAFB test, CD4 count); clinical (other opportunistic infections and duration on HIV treatment).

**Data management and statistical analysis:** data were exported into Stata version 15 (Stata Corp, College Station, TX, USA). Data were checked for errors, outliers and completeness. Frequencies and percentages were used to describe categorical variables while median and inter-quartile ranges (IQR) were used to summarise continuous variables while assuming non-normality. Pearson´s Chi-square test of independence was used to assess presence or otherwise of any significant relationship between exposure variables and outcome variable “level of adherence”. Backward elimination model selection method using p<0.10 as a determinant of significant variables was applied. Variables known apriori to be associated with adherence and those with p<0.10 in bivariate analysis were included in the multivariable logistic regression model to determine factors associated with adherence to clinic appointments. Hosmer and Lemeshow test was used to assess the model´s goodness of fit. All variables with p<0.05 in the multivariable analysis, were considered to be statistically significant.

## Results

As depicted in Table 1, there were 10,427 participants that were involved in this study. Of these, 1,559 (15%) showed good adherence to clinic appointments while 8,868 (85%) adhered poorly. Participants who were co-infected showed poor adherence to clinic appointments 14.2% (p<0.001) with median month on treatment of 39.4 (23.8-60.4). Adherence to clinic appointments decreased with increasing age of the individuals (from 49% in the age group 35-50 to 8.7% in 50+ years), however, this was not statistically significant (p=0.07). Those who were employed had poor level of adherence to clinic appointments, whilst smokers and former smokers had a good level of adherence. There was a significant association between adherence to clinic appointments and WHO stage of HIV diseases at baseline (p<0.001). Adherence to clinic appointments decreased as HIV disease progressed. Individuals with haemoglobin level >11mg/dl adhered more compared to individuals with haemoglobin concentration of <11mg/dl. On univariate analysis, we found that there was a significant association between (being co-infected at baseline, history of alcohol consumption, employment status, history of smoking, haemoglobin level, WHO stage 2, BMI <18, months on treatment, months on treatment by co-infection) and adherence to clinic appointments (Table 2).

**Table 1:** summary of baseline characteristics of patients by adherence to clinic appointments

Variable	Categories	Levels of adherence n/N (%)		p-value
		Poor 8,868(85%)	Good 1,559(15%)	
**Co-infection at baseline**	Yes	1257(14.2)	271(17.4)	0.001
	No	7611(85.8)	1288(82.9)	
**Sex**	Female	5793(65.3)	988(63.4)	0.136
	Male	3075(34.7)	571(36.9	
**Alcohol consumption (n=10065)**	Yes	1118(13)	171(11.3)	0.012
	No	7028(81.7)	1186(81.2)	
	Past history	458(5.3)	104(7.1)	
**Months on treatment, median (IQR)**		39.4(23.8-60.4)	27.8(18.4-43.1)	<0.001*
**Age, median (IQR)**		36.4(31.3-42.7)	35.7(30.6-49.0)	0.004*
**Age group**	18-34	3401(38.4)	645(41.4)	0.07
	35-50	4619(52.1)	778(49.9)	
	50+	848(9.6)	136(8.7)	
**Employment (n=10255)**	Employed	4595(52.6)	631(41.5)	<0.001
	Unemployed	4141(47.4)	888(58.5)	
**Education (8004)**	Illiterate	280(3.9)	31(3.7)	0.166
	Primary	1146(16)	155(18.5)	
	Secondary and beyond	5742(80)	650(77.8)	
**Smoking status (n=10050)**	Yes	954(11.1)	177(12.1)	0.001
	No	7359(85.7)	1209(82.9)	
	Past history	278(3.2)	73(5)	
**Baseline CD4 cell count**	<350	7796(87.9)	1391(89.2)	0.335
	350-500	162(1.8)	25(1.6)	
	>500	910(10.3)	143(9.2)	
**Hemoglobin level**	<11	2837(32.5)	551(35.7)	0.013
	>11	5900(67.5)	992(64.3)	
**WHO stage at day 7 after ART initiation (n=8393)**	1	3019(42.5)	545(42.3)	0.001
	2	1416(19.9)	203(15.7)	
	3	1977(27.8)	405(31.4)	
	4	692(9.7)	136(10.6)	
**BMI (n=10352)**	<18.5	1168(13.3)	266(17.2)	<0.001
	18.5-24.9	4134(47)	738(47.6)	
	25.0-29.9	1285(14.6)	185(11.9)	
	≥30	2216(25.2)	360(23.2)	

* Mann-Whitney test; COR-crude odds ratio; AOR-adjusted odds ratio; CI-confidence interval

**Table 2:** univariable and multivariable analysis of factors associated with adherence

Variable	Categories	COR (95% CI)	P-value	AOR (95% CI)	p-value
**Co-infection at baseline**	No	Reference	0.001	Reference	<0.001
	Yes	0.78 (0.68,0.91)		0.79(0.68, 0.91)	
**Sex**	Male	Reference	0.136	Reference	0.210
	Female	0.92(0.82,1.03)		0.92(0.81, 1.05)	
**Alcohol consumption**					**0.004**
	No	Reference		Reference	
	Yes	0.91 (0.76,1.08)	0.263	0.81(0.68, 0.97)	0.024
	Past history	1.35 (1.08,1.68)	0.009	1.29(1.02, 1.62)	0.032
**Age group**					**0.041**
	50+	Reference		Reference	
	35-50	1.18 (0.97,1.44)	0.10	1.29(1.04, 1.59)	0.020
	18-34	1.05 (0.86,1.28)	0.625	1.16(0.94, 1.43)	0.171
**Employment**	Unemployed	Reference	<0.001	Reference	<0.001
	Employed	0.64 (0.57,0.72)		0.61(0.55, 0.69)	
**Education**	Secondary and beyond	Reference			
	Primary	1.2(0.99,1.44)	0.061		
	Illiterate	0.98(0.67,1.43)	0.909		
**Smoking status**	No	Reference			
	Yes	1.13(0.95,1.34)	0.165		
	Past history	1.60(1.23,2.08)	0.001		
**Baseline CD4 count**					**<0.001**
	>500	Reference			
	350-500	0.98(0.62,1.16)	0.938	1.16(0.73, 1.87)	0.529
	<350	1.13(0.94,1.37)	0.179	1.48(1.20, 1.82)	0.001
**Haemoglobin**	>11	Reference	0.013		
**Level**	<11	1.16(1.03,1.29)			
**WHO baseline**	4	Reference			
	3	1.04(0.84,1.29)	0.702		
	2	0.73(0.57,0.92)	0.009		
	1	0.92(0.75,1.13)	0.417		
**BMI**	≥30	Reference			
	25.0-29.9	0.89(0.73,1.07)	0.213		
	18.5-24.9	1.10(0.96,1.26)	0.175		
	<18.5	1.40(1.18,1.67)	<0.001		
**Months on treatment**		0.97(0.97,0.98)	<0.001	0.98(0.97, 0.98)	<0.001
**Months on treatment by co-infection**		0.98(0.965,0.99)	<0.001	0.98(0.97, 0.99)	<0.001

COR-crude odds ratio; AOR-adjusted odds ratio; CI-confidence interval

Individuals co-infected at baseline were less likely to adhere to clinic appointments compared to those who were not co-infected at baseline (OR: 0.78, 95% CI: 0.68-0.91). There was also an association between duration in months on treatment and adherence to scheduled clinic appointments among co-infected patients (OR: 0.97, 95%CI: 0.97-0.98). This shows that adherence to clinic appointments decreased by 2% for every single month that the patient stayed on treatment. On multivariable logistic regression, factors associated with adherence to clinic appointments were 'alcohol consumption, age group, employment, baseline CD4 count, months on treatment and months on treatment by co-infection'. Patients co-infected with TB had 21% reduced odds of adherence to clinic appointments compared with patients living with HIV only (AOR: 0.79, 95% CI: 0.68, 0.91). Those who consumed alcohol were 19% less likely to adhere to clinic appointments compared with the non-alcoholics (AOR: 0.81, 95% CI: 0.68, 0.97).

Patients with a previous history of alcohol consumption were approximately 1.3 times more likely to adhere to clinic appointments than patients who were using alcohol (AOR: 1.29, 95% CI: 1.02, 1.62). The odds of adherence to clinic appointments were higher among patients aged 35-50 years compared with patients who were ≥50 years of age (AOR: 1.29, 95% CI: 1.04, 1.59). Patients with employment were less likely to adhere to clinic appointments compared with patients who were unemployed (AOR: 0.61, 95% CI: 0.55, 0.69). Furthermore, patients with much lower immune status as evidenced by a CD4 count of less than 350cell/ml^3^ had increased odds of good adherence to clinic appointments (AOR: 1.48 95% CI: 1.20, 1.82). This is a demonstration of a willpower to recover. In addition, patients who were on treatment for a longer period had decreased odds of adherence to clinic appointments compared with patients who had been on treatment for a shorter period.

## Discussion

This study evaluated determinants of adherence to clinic appointments among patients with TB/HIV co-infection. We found that the longer a patient was on treatment the lower the likelihood of adhering to clinic appointments (AOR: 0.98, 95% CI: 0.97, 0.98). This is essential as it provides a platform for continued adherence counselling among patients on treatment specifically those who have been on treatment for a lengthy period. Our findings are similar to other studies that showed long term treatment compromised patients´ will to keep up with scheduled clinic appointments [21-23]. Missed appointments are a catalyst for poor treatment adherence because patients would have no pills as they missed their pill collection time [13,24,25]. Thus, it would be a catalyst to poor treatment outcomes as their attending physician would be unaware of any ailments the patient could be having as they missed their review date. Proper follow up for clinical assessment as scheduled is an important component of the patient´s rigorous assessment to ascertain if the patients have no underlying illness requiring a different type of medication. Furthermore, patients are assessed on any side effect they might be having, to warrant treatment discontinuation or change of treatment regimen to a different type. All these are best carried out if patients maintained their regular follow up visits as scheduled [5,26]. However, a study of two regional cohorts: sub-Saharan Africa and Asia reported different findings. The longer a patient was on ART, the higher the chances of good adherence. This could have been due to patients´ self-reported adherence being high [27].

Overall, HIV/TB co-infected patients had poor adherence to clinic appointments. A study conducted in South Africa reported similar findings [28]. The reasons for this could be patient related such as high pill burden, adverse reactions and fear of not being able to tolerate both ART and anti-tuberculosis medication [19,28-30]. The site of the TB infection was also associated with adherence. Patients with pulmonary TB were less likely to adhere to clinic visits compared with those with extra pulmonary TB [28]. Our study found that patients who consume alcohol have poor adherence. Similar findings were reported where consumption of alcohol resulted in missed clinic visits and in turn poor medication adherence [5,31]. Shumba *et al*. analysed factors associated with missing ART doses and noted that missing clinic appointments was linked to missing doses [32]. Alcohol consumption may cause cognitive impairment which could result in patients failing to make it for their clinic appointments or forget to take their pills, leading to poor adherence [21,32,33]. Patients with a history of alcohol consumption had good adherence than those who did not consume alcohol. Research conducted in Thailand reported good adherence in former drinkers as well and concluded that the good adherence among former drinkers could possibly be due to patients following the health care workers guidelines on lowering or stopping alcohol consumption to improve treatment outcome [33].

Patients 35-50 years of age had good adherence compared with those aged 50 years and older. Financial constraints could have been a potential reason for the older individuals missing their clinic appointments as noted by another study conducted in South Africa [24]. Patients with employment history adhered poorly compared with the unemployed. This is different from another study that noted employment as having a positive influence on attendance to scheduled clinic visits. Being employed offered individuals financial means to visit the clinic [34]. Our results could be different due to missing data as employment status was not available for all participants. Patients with low clinical values of CD4 count <350 had nearly 1.5 times the odds of good adherence (AOR: 1.48 95% CI: 1.20, 1.82). This could have been due to a clear understanding of the risk associated with low CD4 in HIV infection. This is however in contrast with a study conducted in America which noted that individuals that were very ill were possibly less mobile and thus miss the scheduled clinic visit [35]. Another possible reason for our findings could be that patients with clinically normal values felt healthier and thus less inclined to take their medication [36].

## Conclusion

Duration on treatment had a negative influence on adherence to clinic appointments among HIV/TB co-infected patients in this study. Long term treatment appears to compromise an individual´s will to keep up with scheduled clinic appointments. This is problematic among patients with TB/HIV co-infection as missing appointments can result in poor treatment adherence and in turn poor outcomes.

### What is known about this topic

- Tuberculosis remains the leading opportunistic infection among HIV infected individuals thus the continued rampage of TB/HIV co-infection;
- Adherence to TB/HIV co-infection is still a challenge despite the advances in accessibility of ART;
- Good adherence among TB/HIV co-infected individuals results in a reduction of new HIV cases, decreases occurrences of drug resistance and improves the individuals´ quality of life.

### What this study adds

- Adherence to ART was poor in this study: the longer a patient stayed on treatment, adherence became poor whereas previous studies reported good adherence overtime;
- Factors that influenced adherence negatively among the TB/HIV co-infected patients included 'co-infection at baseline, alcohol consumption, employment status, baseline CD4 count, months on treatment and months on treatment by co-infection';
- Further research needs to be conducted regarding duration on treatment as a negative influence on adherence.
